# The relationship of laryngopharyngeal reflux-related signs and symptoms to vocal complaints in professional classical singers

**DOI:** 10.3325/cmj.2026.67.264

**Published:** 2026-08

**Authors:** Marin Čargo, Željka Laksar Klarić, Ana Đanić Hadžibegović

**Affiliations:** 1 Faculty of Health Studies, Catholic University of Croatia, Zagreb, Croatia; 2 Department of ENT and Head and Neck Surgery, Faculty of Medicine Osijek, University Hospital Centre Osijek, Osijek, Croatia; 3 Otorhinolaryngology Clinic, Department of Phoniatrics, University Hospital Center Zagreb, Zagreb, Croatia; 4 Faculty of Dental Medicine and Health, University J.J. Strossmayer, Osijek, Croatia

## Abstract

**Aim:**

To examine the relationship between laryngopharyngeal reflux-related signs, symptoms, and self-reported vocal complaints in professional classical singers.

**Methods:**

This cross-sectional study enrolled 111 classically trained singers. Participants completed a questionnaire on vocal habits and complaints, as well as the validated Reflux Symptom Score (RSS). Data were collected at the phoniatrics departments of the University Hospital Center Zagreb and the University Hospital Center Osijek in 2023. LPR signs were assessed using flexible nasopharyngolaryngoscopy and the Reflux Sign Assessment (RSA). Spearman’s rank correlation, multivariable regression, and multivariate analysis of variance were used to examine associations according to RSA and RSS cutoff values.

**Results:**

A total of 29 (26.13%) participants exceeded the RSA cutoff value. Fifty-seven (51.35%) exceeded the RSS cutoff. RSA and RSS total scores were weakly but significantly positively correlated. The total RSS score emerged as a significant predictor of higher RSA scores, although the explained variance was modest. Compared with singers who did not exceed the RSA or RSS cutoff values, singers exceeding RSA or RSS cutoff values demonstrated a different pattern of vocal complaints, with more frequent throat clearing, hoarseness, and other vocal disturbances, vocal fatigue, and post-performance deterioration of voice quality.

**Conclusion:**

LPR-related signs and symptoms were common in professional classical singers, with subjective symptom burden exceeding objective laryngoscopic findings. The weak concordance between signs and symptoms highlights the multifactorial nature of voice complaints in this population. These findings support an integrated approach combining reflux-oriented management with targeted voice therapy rather than reliance on isolated laryngoscopic findings.

Laryngopharyngeal reflux (LPR) is an inflammatory disease of the upper aerodigestive tract caused by backflow of gastric contents into the pharynx and larynx, leading to morphological tissue changes (1,2). The symptoms are often nonspecific and may include throat discomfort, post-nasal drip, throat clearing, dysphagia, heartburn, regurgitation, and hoarseness (3-5).

Typical laryngeal findings are endolaryngeal mucus, arytenoid inflammation, and posterior commissure hypertrophy (PCH), while extra-laryngeal findings include hypo-pharyngeal and oropharyngeal erythema, pharyngeal granulations, uvula erythema or edema, and anterior pillar erythema (3,6).

LPR affects up to 55% of patients with hoarseness and is frequently associated with vocal problems in classically trained singers, including opera soloists and students already involved in performances (1,3,4,7). Even minimal alterations of the vocal fold epithelium and superficial lamina propria can lead to significant vocal restrictions in this population (3). Pepsin-mediated epithelial microtrauma may therefore contribute to hoarseness, vocal fatigue, and range loss among singers.

The technique of supported voice (breath support, *appoggio*) increases intra-abdominal pressure and load on the lower esophageal sphincter (LES), particularly during repeated deep inspirations and prolonged phonation, potentially contributing to LES dysfunction and an increased risk of LPR (1). Stress related to career demands and frequent late meals after performance may further predispose singers to reflux (3).

Because any voice problem directly affects both the livelihood and emotional well-being of professional singers, a healthy laryngeal mechanism is essential for a successful and sustained career (8,9). In a recent systematic review, Lechien et al (10) highlighted the need for studies on laryngopharyngeal reflux in singers that combine validated symptom instruments with structured laryngoscopic evaluation in well-defined singer cohorts. The aim of this study was to investigate the relationships of LPR-related signs and symptoms and voice complaints in professional classical singers.

## Participants and methods

### Participants

Data were collected at the phoniatrics departments of the University Hospital Center Zagreb and the University Hospital Center Osijek in 2023. The participants were recruited by contacting Croatian National Theatres in Zagreb and Osijek, the University of Zagreb Academy of Music, and the Croatian Radio and Television Choir. These institutions distributed invitations to professional classically trained singers and early-stage professionals already involved in professional performances. Participants were also encouraged to share information about the study with other eligible colleagues. The inclusion criterion was regular performing or study activity, while the exclusion criterion was an acute upper respiratory disease immediately before or at the time of examination.

The study was conducted in accordance with the Declaration of Helsinki and approved by the Ethics Committee of the University Hospital Center Zagreb and the University Hospital Center Osijek. Participants signed an informed consent form before participation.

### Data collection

Participants completed a 14-item questionnaire covering sociodemographic data (age, sex), body weight and height, vocal hygiene habits, vocal complaints, and medication use. Body mass index (BMI) was calculated by one of the researchers. The questionnaire was developed by the authors based on existing vocal hygiene literature and clinical experience. Items were rated on a 5-point Likert scale (1 = never, 5 = every day).

### Phoniatric examination and a Reflux Sign Assessment scale

For an objective assessment of LPR, participants underwent a complete phoniatric examination, including oropharyngeal examination, anterior rhinoscopy, and flexible nasopharyngolaryngoscopy (Olympus CV-180 EVIS EXERA II, Olympus, Tokyo, Japan). During endoscopy, participants were asked to produce a sustained vowel “i” at habitual pitch and glissando. For each participant, the Reflux Sign Assessment (RSA) scale was completed by a phoniatrician. Examinations were performed by two experienced phoniatric otorhinolaryngologists, and the RSA and its short form have demonstrated good to excellent reliability (5,11). The RSA comprises oral, pharyngeal, and laryngeal items. An RSA score >14 may suggest LPR (5). However, it does not establish a definitive diagnosis in the absence of objective reflux testing.

### Reflux Symptom Score questionnaire

For subjective assessment of LPR, participants completed the Reflux Symptom Score (RSS) questionnaire. The RSS covers ear, nose, and throat, abdominal, and respiratory symptoms. The frequency and severity of each symptom are rated on a 6-point Likert scale. For each item, a composite symptom score (from 0 to 25) is obtained by multiplying severity and frequency. An RSS score >13 suggests LPR-related symptom burden (6). In this study, we did not use the quality of life impact items of RSS.

### Statistical analysis

Ordinal data are expressed as frequencies and percentages, while continuous data are expressed as means and standard deviations. Correlations between continuous or ordinal variables were assessed using Spearman’s rank correlation coefficient. To identify independent predictors of LPR, multivariable binary logistic regression models were fitted with RSA and RSS cutoff status as dependent variables and sociodemographic and lifestyle variables as predictors. In addition, a multivariable linear regression model was fitted with the total RSA score as the continuous dependent variable and the total RSS score and age as predictors, to examine whether symptom burden predicted the severity of laryngoscopic findings. MANOVA was used to compare the overall pattern of self-reported vocal complaints between participants with and without LPR, followed by univariate tests for individual complaints. A *P* value <0.05 was considered statistically significant. The statistical analysis was performed with SPSS, version 26 (IBM Corp., Armonk, NY, USA).

## Results

### Socio-demographic data

The study included 111 participants: 69 professional singers and 42 early-stage professionals. The mean age was 35.69 years (SD 12.06), with 57.7% of participants being women. The mean BMI was 25.77 (SD 4.39).

### Vocal hygiene habits, vocal complaints, and medication use

Although infrequent, tobacco consumption was the only habit that was weakly but significantly negatively correlated with RSS scores (ρ = −0.19, *P* < 0.05). Other lifestyle habits were moderately prevalent but were not significantly correlated with RSA or RSS scores.

Vocal muscle fatigue was weakly positively correlated with RSA scores (ρ = 0.19, *P* < 0.05) and moderately positively with RSS scores (ρ = 0.32, *P* < 0.01). The perception of poorer voice quality after a rehearsal or performance was weakly to moderately correlated with RSS scores (ρ = 0.28, *P* < 0.01) but not with RSA. Hoarseness, vocal noise, or voice “crackling/breaking” was weakly positively correlated with RSA (ρ = 0.22, *P* < 0.05) and moderately positively with RSS (ρ = 0.37, *P* < 0.01). The use of anti-reflux medications (proton pump inhibitors and/or alginates) was low (6.3%) (Table 1).

**Table 1:** The correlations (Spearman’s ρ correlation coefficient) between vocal hygiene habits, vocal complaints, medication use, and Reflux Sign Assessment scale (RSA) and Reflux Symptom Score (RSS) scale results

Vocal hygiene habits	M	SD	ρ RSA	ρ RSS
I consume tobacco products	1.70	1.15	0.03	−0.19^†^
I eat my last meal less than two hours before going to bed	2.90	1.12	−0.09	0.00
My last meal is of liquid consistency	2.32	0.97	0.00	−0.05
I consume alcohol	2.59	1.03	−0.08	−0.04
I consume carbonated drinks	2.59	0.96	0.14	0.09
**Vocal complaints**				
I experience symptoms of vocal muscle fatigue during or after singing (tension in the neck and shoulders, a feeling of a constricted throat)	2.32	0.85	0.19^†^	0.32*
After a rehearsal/performance, I feel that my voice is not of the same quality as at the beginning or before the rehearsal/performance	2.47	0.88	0.04	0.28*
My vocal range is reduced after a rehearsal/performance	1.93	0.95	0.11	0.12
I experience issues such as hoarseness or noise in the voice, as well as “crackling” or “breaking” of the voice	2.00	1.00	0.22^†^	0.37*
**Medication use**				**N (%)**
Use of proton pump inhibitors and/or alginates				7 (6.3)

*P < 0.01.

†*P* < 0.05.

### Laryngoscopic examination and the Reflux Sign Assessment scale

Seventeen of 69 professional singers (24.63%) and 12/42 early-stage professionals (28.57%) exceeded the RSA cutoff suggestive of LPR. Reflux signs were equally frequent in both subgroups (χ^2^_RSA(1)_ = 0.21, *P* = 0.65). The most common pathological findings identified by the phoniatrician during examinations in the entire group were anterior pillar erythema (41.4%), PCH (39.6%), and erythema of arytenoids/inter-arytenoid (37.8%) (Table 2).

**Table 2:** Reflux Sign Assessment scale results

Reflux Sign Assessment	No. (%)
Anterior pillar erythema	46 (41.4)
Uvula erythema ± edema	22 (19.8)
Coated tongue	4 (3.6)
Nasopharyngeal wall erythema ± inflammatory granulations	10 (9.0)
Posterior oro- or hypopharyngeal wall erythema	17 (15.3)
Posterior oro- or hypopharyngeal wall inflammatory granulations	26 (23.4)
Tongue tonsil hypertrophy – apparent vallecula only when the tongue is stuck out	20 (18)
Tongue tonsil hypertrophy – unapparent vallecula irrespective the tongue	11 (9.9)
Contact between epiglottis and tongue tonsils	9 (8.1)
Pharyngeal sticky mucus	19 (17.1)
Subglottic edema ± erythema	2 (1.8)
Ventricular band erythema ± edema	7 (6.3)
Epiglottis redness ± edema	2 (1.8)
Erythema – arytenoids/inter-arytenoid only	42 (37.8)
Erythema – diffuse to posterior commissure	7 (6.3)
Inter-arytenoid granulatory tissue	8 (7.2)
Posterior commissure hypertrophy	44 (39.6)
Retro-cricoid erythema	7 (6.3)
Retro-cricoid edema	8 (7.2)
Endolaryngeal sticky mucus deposit	21 (18.9)
Vocal fold erythema	7 (6.3)
Edema of the free-edge or the entire vocal folds	1 (0.9)
Vocal fold lesions: nodules	3 (2.7)

### Reflux Symptom Score questionnaire

Overall, 35/69 professional singers (50.72%) and 22/42 early-stage professionals (52.38%) exceeded the RSS threshold suggestive of LPR. Reflux symptoms were equally frequent in both subgroups (χ^2^_RSS(1)_ = 0.03, *P* = 0.86). When it came to subjective assessment of reported reflux symptoms, the participants in the entire group most often reported excess mucus in the throat or post-nasal drip sensation (72.1%), abdominal distention and/or flatus (67.6%), clearing the throat (65.8%), heartburn, stomach acid coming up (54.1%), regurgitations of liquids, solid foods, or burps (53.2%), hoarseness or a voice problem (48.6%), and sensation of something sticking in the throat (47.7%) (Table 3). Since professional singers and early-stage professionals did not differ in the proportion of participants exceeding RSA and RSS cutoff values, subsequent analyses and interpretations are based on the total sample.

**Table 3:** Reflux Symptom Score (RSS) questionnaire results

Disorder type	No. (%)
Ear, nose, throat disorders	
Hoarseness or a voice problem	54 (48.6)
Throat pain	39 (35.1)
Pain during swallowing time	27 (24.3)
Difficulty swallowing (pills, liquids or solid foods)	16 (14.4)
Clearing the throat	73 (65.8)
Sensation of something sticking in the throat	53 (47.7)
Excess mucous in the throat or post-nasal drip sensation	80 (72.1)
Ear pressure/pain (daytime or night-time)	10 (9)
Tongue burning	14 (12.6)
**Abdominal disorders**	
Heartburn, stomach acid coming up	60 (54.1)
Regurgitations of liquids, solid foods, or burps	59 (53.2)
Abdominal pain	43 (38.7)
Diarrhea	44 (37.8)
Constipation	22 (19.8)
Indigestion	42 (37.8)
Abdominal distension and/or flatus	75 (67.6)
Halitosis	43 (38.7)
Nausea	36 (32.4)
**Chest/respiratory disorders**	
Cough after eating or lying down	18 (16.2)
Cough (daytime)	37 (33.3)
Breathing difficulties, breathlessness, or wheezing	28 (25.2)
Chest pain	16 (14.4)

### Correlation of RSA and RSS with BMI and age

RSA and RSS scores were weakly positively correlated (ρ = 0.31, *P* < 0.05), while BMI and age were moderately correlated (ρ = 0.53, *P* < 0.01); sex was not significantly correlated with either RSA or RSS scores (Table 4).

**Table 4:** Spearman's ρ correlation coefficients between Reflux Symptom Assessment (RSA) and Reflux Symptom Score (RSS) with body mass index (BMI) and age

	RSA	RSS	BMI	Age	Sex
1. RSA	1	0.31^†^	−0.02	−0.13	−0.01^‡^
2. RSS	-	1	−0.01	−0.01	0.12^‡^
3. BMI	-	-	1	0.53*	−0.31*^‡^
4. Age	-	-	-	1	−0.08^‡^
5. Sex	-	-	-	-	1

*P < 0.01.

†*P* < 0.05.

‡Spearman's ρ correlation coefficient.

A multivariable linear regression model with the total RSA score as the dependent variable and the total RSS score and age as predictors was significant (F(2,108) = 6.71, *P* = 0.002), explaining approximately 11% of the variance in RSA scores (R^2^ = 0.11, adjusted R^2^ = 0.09). The total RSS score was a significant positive predictor of the RSA score (B = 0.09, SE = 0.03, β = 0.31, *P* < 0.001), whereas age was not significantly associated with RSA after adjustment (*P* = 0.179) (Supplementary Table 1(Supplementary Table 1)).

### Multivariable analysis of predictors of LPR

Exploratory logistic regression analyses suggested a possible association between higher carbonated beverage consumption and LPR-related signs, but estimates were imprecise and unstable, and should therefore be interpreted as hypothesis-generating only (Supplementary Table 2(Supplementary Table 2)). A corresponding multivariable logistic regression model with the RSS cutoff as the dependent variable was also fitted but did not reach statistical significance overall, and no individual predictor was significantly associated with RSS-defined LPR. These results are therefore not presented in detail.

### Vocal complaints according to RSA and RSS results

To directly compare the discriminative ability of the RSA and RSS cutoff values, the results of both MANOVA and univariate analyses are presented together. At the multivariate level, the RSS classification demonstrated a stronger overall effect on the combined set of vocal complaints (Pillai's Trace; *F*(5,105) = 6.783, *P* < 0.001) compared with the RSA classification (*F*(5,105) = 2.513, *P* = 0.034). At the univariate level, the RSS cutoff value significantly differentiated groups in four out of five items (vocal muscle fatigue, voice disturbances, throat clearing, and voice quality; all *P* ≤ 0.002), whereas the RSA cutoff value differentiated groups in only two items (voice disturbances and throat clearing; *P* = 0.032 and *P* = 0.003). Neither classification showed a significant difference in reduced vocal range (*P* = 0.157 for RSS; *P* = 0.485 for RSA) (Table 5).

**Table 5:** Multivariate and univariate analysis of self-reported vocal complaints according to Reflux Symptom Assessment (RSA) and Reflux Symptom Score (RSS) cutoff values

	RSA cutoff F (df1, df2)	RSA p	RSS cutoff F (df1, df2)	RSS p
Multivariate (MANOVA, Pillai; 5 DV)	2.513 (5, 105)	0.034	6.783 (5, 105)	<0.001
I experience symptoms of vocal muscle fatigue during or after singing (tension in the neck and shoulders, a feeling of a constricted throat)	1.354 (1, 109)	0.247	11.38 (1, 109)	0.001
My vocal range is reduced after a rehearsal/performance	0.491 (1, 109)	0.485	2.035 (1, 109)	0.157
I experience issues such as hoarseness or noise in the voice, as well as “crackling” or “breaking” of the voice	4.740 (1, 109)	0.032	9.792 (1, 109)	0.002
Need to clear the throat	9.296 (1, 109)	0.003	27.86 (1, 109)	<0 .001
After a rehearsal/performance, I feel that my voice is not of the same quality as at the beginning or before the rehearsal/performance	0.010 (1, 109)	0.920	10.27 (1, 109)	0.002

## Discussion

In this study, LPR-related signs and symptoms were common within a cohort of classical professional singers. Approximately one quarter of participants demonstrated laryngoscopic findings suggestive of LPR according to RSA criteria, whereas approximately one half reported symptoms suggestive of LPR according to RSS. This suggests that subjective complaints were more common than endoscopic signs of reflux. Although RSA and RSS were significantly positively correlated, the strength of the correlation was low.

### RSA and RSS findings

Gelardi et al (12) reported a moderate positive correlation between subjective symptoms and objective laryngoscopic findings in patients with LPR, whereas in our study this correlation was weaker. The observed weak correlation warrants careful interpretation. Multivariable linear regression analysis was consistent with this pattern, showing that the total RSS score remained a significant positive predictor of the total RSA score after adjustment for age. However, the model explained only 11% of the variance in RSA, indicating that symptom burden and morphological signs captured only partially overlapping aspects of LPR in this population. This finding is consistent with previous observations that, in LPR assessment, laryngoscopic findings do not always correlate strongly with patient-reported symptoms (12,13).

Several mechanisms possibly account for this discordance: professional voice users may recognize symptoms before structural changes become endoscopically visible; transient reflux episodes may elicit immediate symptoms such as excess mucus and throat clearing without producing lasting laryngeal alterations; and many laryngoscopic findings attributed to LPR are relatively nonspecific. In addition, some complaints may arise from other causes such as vocal muscle fatigue, allergy, dehydration, or co-occurring conditions common in singers, underscoring the need for careful differential diagnosis rather than attributing all voice problems to LPR (8,14). The weak negative correlation between tobacco use and RSS likely reflects restriction of range, as the vast majority of participants reported minimal smoking.

Nacci et al (15) reported laryngoscopic signs of LPR in 22.6% of 159 singers, closely mirroring our finding that roughly one quarter of participants exceeded the RSA cutoff.

Among the most frequent pathological findings observed in our study were anterior pillar erythema and PCH. Anterior pillar erythema is a highly sensitive sign of LPR, whereas PCH, although highly sensitive, demonstrates markedly low specificity (16-18). Recent evidence shows that PCH may persist after successful acid suppression, suggesting chronic remodeling rather than active reflux, and it poorly correlates with symptom severity (19). PCH in singers may partly reflect cumulative biomechanical stress of intense phonation, a hypothesis warranting further investigation. Erythema of the arytenoids/inter-arytenoid region was also common and is often associated with chronic inflammatory changes, including LPR (20,21).

In this study, half of the participants subjectively reported symptoms compatible with LPR. In a similar study (22), including 392 amateur choir singers, 11.5% had a Reflux Symptom Index total score above the cutoff. Choir singers expressed more complaints related to their voice and the vocal tract.

The most frequently reported LPR symptoms from the group of ear, nose, and throat symptoms in our study were excess mucus in the throat or post-nasal drip sensation and throat clearing. Gastrointestinal symptoms included abdominal distension and/or flatus, regurgitation of liquids, solid foods, or burps, heartburn, and stomach acid coming up. Nearly half of the participants reported hoarseness or other voice problems. Throat clearing is a form of phonotraumatic behavior that can mechanically damage the vocal folds through repeated forceful contact and collision forces higher than those during normal phonation (14). A likely explanation is that acid reflux irritates the laryngeal mucosa frequently triggering reflexive throat clearing and coughing, both of which increase the risk of mechanical damage to the vocal folds (1). Excess mucus may represent a mucosal defense response to pepsin-mediated microtrauma and acid-induced dehydration (4).

Abraham et al (13) reported remarkably similar results to ours: the most common symptoms were globus sensation, pooling of mucus in the throat, and frequent throat clearing, and the most common signs were hyperemia/erythema of the endolarynx, thick endolaryngeal mucus, and PCH. The authors (13) also suggested combining subjective and objective assessment for LPR, as was done in the present study.

According to Lechien et al (16), throat clearing, heartburn, and excess throat mucus, particularly in combination, show high sensitivity and negative predictive value for symptoms and signs of LPR. This is further supported by our regression findings, in which RSS explained only approximately 11% of RSA variance. This modest shared variance is consistent with previous reports of limited concordance between symptom questionnaires and laryngoscopic findings in LPR.

In addition, exploratory logistic regression suggested a possible association between higher carbonated beverage consumption and RSA-defined LPR, but this effect was characterized by very wide confidence intervals and disappeared when the exposure was dichotomized. Accordingly, this signal should be regarded as hypothesis-generating and interpreted with caution until replicated in larger cohorts. The instability of this estimate is consistent with the low ratio of outcome events to predictors in the model.

### Vocal complaints and clinical implications

Hoarseness, reported by nearly half of our participants, is a well-recognized manifestation of LPR, thought to result from alterations in the biomechanical properties of the vocal folds (3). In our study, however, the rarity of pathological findings on the vocal folds themselves suggests that a substantial subjective impact can occur in the presence of a relatively modest structural change. Experimental research in animals indicates that healthy vocal fold epithelium may be fairly resilient to limited acid-pepsin exposure (23), which is broadly consistent with this pattern, although such findings cannot be directly generalized to humans.

A recent meta-analysis (24) showed clinically significant voice disturbances in patients with LPR. It also showed that voice therapy had a greater effect on voice improvement than proton pump inhibitor therapy alone, while objective voice measures appeared more reliable than symptom-only questionnaires. In this context, the low rate of anti-reflux medication use in our cohort is not necessarily concerning and supports an approach that combines reflux-oriented management with specific voice therapy and objective monitoring rather than relying solely on pharmacological treatment.

The MANOVA results provide further insight into how reflux signs and symptoms relate to vocal complaints in classical singers. The RSA score was significantly associated only with increased throat clearing, hoarseness, and other vocal disturbances, whereas the RSS score showed a broader pattern of associations, including vocal fatigue, post-performance deterioration of voice quality, hoarseness, and other vocal disturbances, and an increased need to clear the throat. These findings suggest that subjective reflux burden is more closely aligned with perceived vocal handicap than with isolated laryngoscopic signs, which is clinically relevant when counseling singers who present with prominent symptoms in the absence of marked morphological changes.

An additional consideration is that RSS scores and vocal complaints were both self-reported. Consequently, the stronger associations observed between RSS positivity and vocal complaints may partly reflect shared-method variance rather than a purely stronger clinical relationship. Therefore, these findings should be interpreted as evidence of association rather than as superiority of symptom-based assessment over laryngoscopic findings.

The strengths of this study are its focus on a hard-to-reach population of professional classically trained singers and its being the first study in Croatia to address this specific group. The exclusive inclusion of singers and the combined use of validated RSS and RSA instruments distinguish it from many previous studies that relied on non-validated questionnaires or symptoms alone (10).

The diagnostic precision of the study is limited by not using invasive methods such as 24-hour pH-metry and multichannel intraluminal impedance. However, singers' ongoing commitments and documented reluctance to undergo such procedures made their use impractical. Recent literature also reports singers’ fear, describing their implementation as challenging (10). Furthermore, RSA and RSS cutoff values in the present study should be interpreted as indicating findings and symptoms suggestive of LPR rather than confirming the diagnosis of LPR. Inter-rater reliability of RSA scoring was not formally assessed; however, examinations were performed by two experienced phoniatric otolaryngologists who adhered strictly to itemized RSA criteria, and previous validation studies have demonstrated good inter-rater reliability for both the original RSA and its short form (5,11). The relatively small sample reflects the limited size of the professional classical singer population in Croatia and may restrict generalizability, while the cross-sectional design and one-month recall period in the questionnaire preclude causal inference and may introduce recall bias. Furthermore, the absence of a non-singer control group limits conclusions regarding whether the observed frequency of LPR-suggestive findings differs from that in the general population. Given the exploratory nature of secondary analyses and the number of statistical comparisons performed, no correction for multiple testing was applied; therefore, *P* values close to the conventional threshold should be interpreted with caution. In the multivariable logistic regression model predicting RSA-defined LPR, the ratio of outcome events to candidate predictors was low, so this model should accordingly be regarded as exploratory and hypothesis-generating rather than confirmatory. Because participation was voluntary, self-selection bias cannot be excluded.

In conclusion, LPR-related signs and symptoms were common in professional classical singers, with subjective symptom burden exceeding objective laryngoscopic findings. The weak concordance between signs and symptoms highlights the multifactorial nature of voice complaints in this population. These findings support an integrated approach combining reflux-oriented management with targeted voice therapy rather than reliance on isolated laryngoscopic findings.
