# Supplementary material for: The relationship of laryngopharyngeal reflux-related signs and symptoms to vocal complaints in professional classical singers
Source: Croat Med J. 2026 Aug;67(4):264–72. doi: 10.3325/cmj.2026.67.264 (PMC13552773; doi:10.3325/cmj.2026.67.264)
Supplement: Supplementary file 1 [file CroatMedJ_67_s001.pdf]

Supplementary Table 1. Multivariable linear regression predicting RSA total score from RSS total score and age

Predictor	B	SE B	β	t	p
Intercept	12.21	2.86	—	4.27	< 0.001
RSS total score	0.09	0.03	0.31	3.38	< 0.001
Age	−0.10	0.07	−0.12	−1.35	0.179

$R = .33$, $R^2 = .11$

$F(2, 108) = 6.71$, $p = .002$, RMSE = 9.28.
