# Supplementary material for: The relationship of laryngopharyngeal reflux-related signs and symptoms to vocal complaints in professional classical singers
Source: Croat Med J. 2026 Aug;67(4):264–72. doi: 10.3325/cmj.2026.67.264 (PMC13552773; doi:10.3325/cmj.2026.67.264)
Supplement: Supplementary file 2 [file CroatMedJ_67_s002.pdf]

Supplementary Table 2. Multivariable logistic regression analysis of predictors of RSA

Predictor	OR	95% CI	p-value
Age (years)	0.95	0.90 – 1.01	0.086
Body mass index (kg/m²)	1.10	0.95 – 1.27	0.224
Sex (male vs female)	0.37	0.12 – 1.10	0.074
Last meal < 2 h before sleep			
Rarely	0.13	0.01 – 1.22	0.075
Occasionally	0.20	0.02 – 1.73	0.145
Often	0.39	0.05 – 3.26	0.385
Alcohol consumption			
Rarely	0.30	0.05 – 1.76	0.180
Occasionally	0.25	0.05 – 1.27	0.094
Often	1.18	0.18 – 7.92	0.862
Carbonated beverage consumption			
Rarely	**11.14**	**1.30 – 95.45**	**0.028**
Occasionally	6.70	0.88 – 51.05	0.066
Often	**13.42**	**1.39 – 130.07**	**0.025**
Model fit<br>$\chi^2 = 28.34$, $p = .020$<br>Nagelkerke $R^2 = .33$			

Note. *The reference category was "Never" for last meal <2 h before sleep, alcohol consumption, and carbonated beverage consumption.*
